# Supplementary material for: Facilitating alkaline hydrogen evolution kinetics via interfacial modulation of hydrogen-bond networks by porous amine cages
Source: Nat Commun. 2025 Feb 21;16:1849. doi: 10.1038/s41467-025-56962-z (PMC11845474; doi:10.1038/s41467-025-56962-z)
Supplement: Supplementary file 1 — Supplementary Information [file 41467_2025_56962_MOESM1_ESM.pdf]

Facilitating Alkaline Hydrogen Evolution Kinetics via Interfacial Modulation of Hydrogen-Bond Networks by Porous Amine Cages

Shiqi Zhou[1], Wei Cao[2], Lu Shang[3], Yunxuan Zhao[3], Xuyang Xiong[4], Jianke Sun[5], Tierui Zhang[3, 6, *], Jiayin Yuan[1, *]

[1] Department of Materials and Environmental Chemistry, Stockholm University, Stockholm 10691, Sweden
[2] Frontiers Science Center for Rare Isotopes, Lanzhou University, Lanzhou 730000, China
[3] Key Laboratory of Photochemical Conversion and Optoelectronic Materials, Technical Institute of Physics and Chemistry, Chinese Academy of Sciences, Beijing 100190, P. R. China
[4] Institutes of Physical Science and Information Technology, Anhui University, Hefei 230601, P. R. China
[5] MOE Key Laboratory of Cluster Science, Beijing Key Laboratory of Photoelectronic/Electrophotonic Conversion Materials, School of Chemistry and Chemical Engineering, Beijing Institute of Technology, Beijing 102488, P. R. China
[6] Center of Materials Science and Optoelectronics Engineering, University of Chinese Academy of Sciences, Beijing 100049, P. R. China

*Correspondence and requests for materials should be addressed to Tierui Zhang (tierui@mail.ipc.ac.cn) or Jiayin Yuan (jiayin.yuan@mmk.su.se)

Table of Contents

Supplementary Methods
Supplementary Figure 1-23
Supplementary Table 1-5
Supplementary References

Supplementary Methods

Climbing-image nudged elastic band calculation

The climbing-image nudged elastic band (CI-NEB) calculation method was applied for the more accurate finding of saddle points here[1,2]. The transition states of Volmer and Heyrovsky steps were searched using CI-NEB method in this work. Three images were inserted into the initial and final configurations for finding the accurate transition states.

HER can be expressed as following equation:

$$2H_2O(l) \rightarrow H_2(g) + 2OH^- \quad (0)$$

The HER activities on active sites were studied in details. HER occur over the active sites under alkaline condition in the following electron-transfer Volmer and Heyrovsky paths according to our experimental Tafel slope results:

$$\text{Volmer step: } H_2O(l) + e^- + {}^* \rightleftharpoons {}^*H + OH^- \quad (1)$$

$$\text{Heyrovsky or Tafel step: } {}^*H + H_2O(l) + e^- \rightleftharpoons H_2(g) + OH^- + {}^* \quad (2)$$

(* indicates the adsorbing site on the catalyst surface)

Where * stands for an active site on the catalyst surface, *(l)* and *(g)* refer liquid and gas phases, respectively, and H* was adsorbed hydrogen atom.

In order to obtain the rate-determining step of HER on different catalysts, we calculated the adsorption free energy of H*, according to the following equation:

$$\Delta E{*}H = E(H{*}) - E({*}) - 1/2E_{H_2} \quad (3)$$

Where E(H*), E(*) and E_{H2} were the ground state energies of surfaces adsorbed with H*, clean surface and H_2 molecules in the gas phase, respectively. we also considered the ZPE and entropy corrections here. These calculations transform DFT binding energies, ΔE^{DFT}, into free energies of adsorption, ΔG_{ads}, by the following equation:

$$\Delta G_{ads} = \Delta E^{DFT} + \Delta ZPE - T\Delta S \quad (4)$$

where T is the temperature and ΔS is the entropy change. For the zero point energy (ZPE), the vibrational frequencies of adsorbed species were calculated with the Pt(100) and Cage-fixed to obtain ZPE contribution in the free energy expression.

For each step, the reaction free energy ΔG is defined as the difference between free energies of the initial and final states and is given by the expression:

$$\Delta G = \Delta E + \Delta ZPE - T\Delta S + \Delta G_U + \Delta G_{pH} \quad (5)$$

where ΔE is the reaction energy of reactant and product molecules adsorbed on the surface of catalyst, obtained from DFT calculations, ΔG_U = -eU, where U is the potential at the electrode, and e is the charge transferred. ΔG_{pH} was the correction of the H^+ free energy by the concentration dependence of the entropy:

$$\Delta G_{pH} = -k_B T ln[H^+] \quad (6)$$

where k_B is the Boltzmann constant.

The free energy of reaction (1) and (2) can be calculated using equation (5).
The solvation effect has been included by VASP sol++, which implements an implicit solvation model that describes the effect of electrostatics, cavitation, and dispersion on the interaction between a solute and solvent[3]. The relative dielectric constant was set to 78.4, corresponding to water at room temperature.
Following the Volmer step, Heyrovsky step was chosen as the second elementary step according the Tafel slope values in our experimental results (Figure 2f) which revealed that the hydrogen evolution reaction becomes Heyrovsky-limited on Pt/cage under alkaline condition.

Calculation of theoretical overpotential

The theoretical overpotentials were analyzed based on the methods reported before[4-7]:

$$G^{HER} = max \Delta G_1, \Delta G_2 \quad (7)$$

$$\eta^{HER} = G^{HER}/e - 0.765V \quad (8)$$

Where ΔG_1 and ΔG_2 are the differences of the energies of reaction (7) and (8), respectively. an ideal catalyst should be able to facilitate the HER just above the equilibrium potential, but requires all the two electron-transfer steps to have reaction free energies of the same magnitude at zero potential (i.e., 1.53 eV/2 = 0.765 eV.) this is equivalent to all the reaction free energies being zero at the equilibrium potential, 0.765V. the reaction energy of the reaction (0) in the alkaline environment can be obtained as 1.53 eV.

Supplementary Figures:

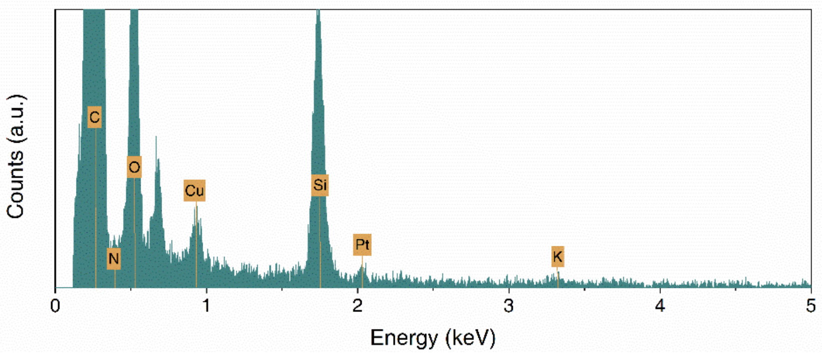

Supplementary Figure 1. EDS spectrum of Pt/cage. Cu comes from the Cu TEM grid.

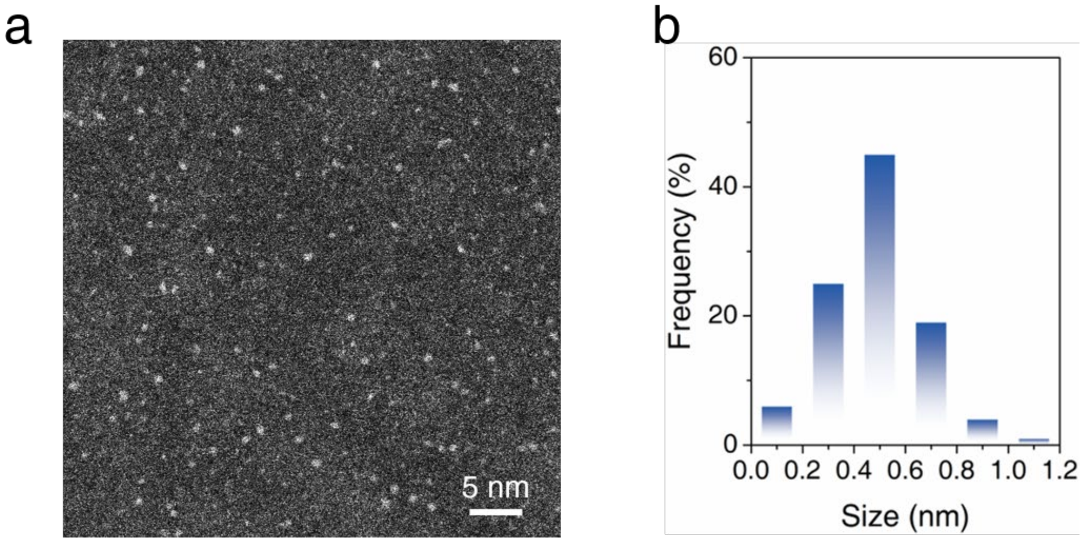

Supplementary Figure 2. (a) HAADF-STEM image and (b) size distribution histogram of Pt clusters confined in porous cage.

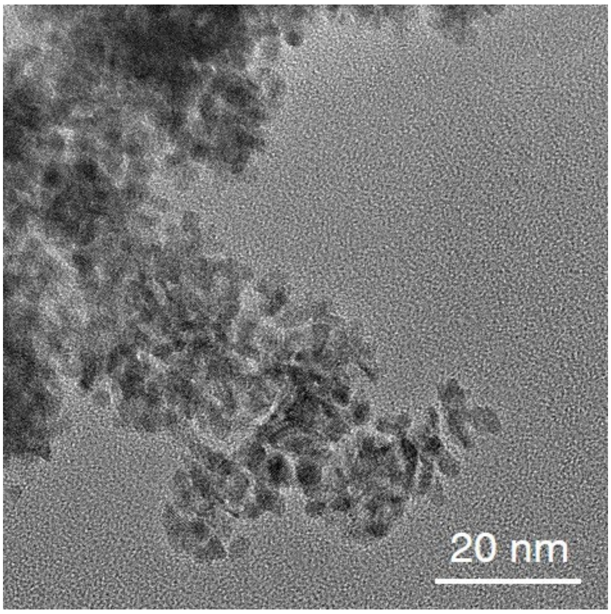

Supplementary Figure 3. TEM image of the as-prepared Pt clusters without adding cage (the average size is 2.5 ± 0.3nm).

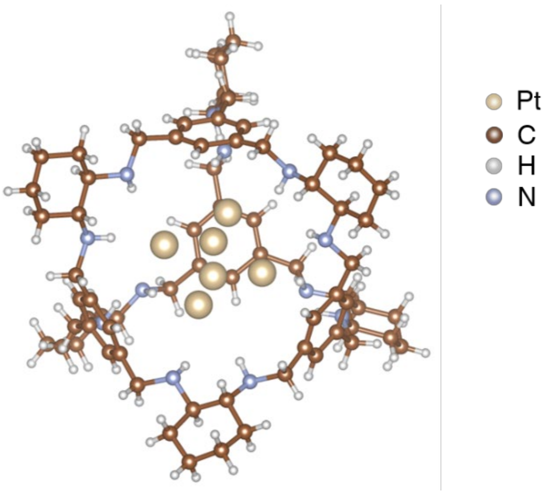

Supplementary Figure 4. Configurational illustration of cage-confined Pt cluster. The corresponding colors for platinum atoms, carbon atoms, hydrogen atoms, and nitrogen atoms are yellow, brown, white, and blue, respectively.

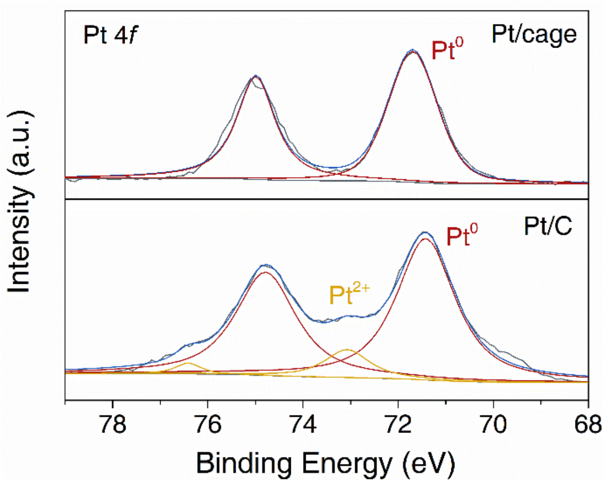

Supplementary Figure 5. X-ray photoelectron spectra of Pt/cage and commercial Pt/C.

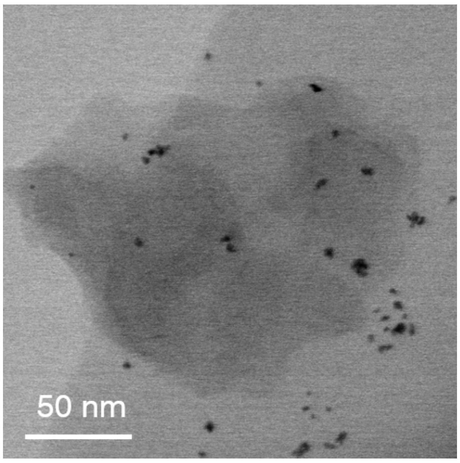

Supplementary Figure 6. HAADF-STEM image of Pt/cage supported on graphite nanoplatelets after removing cage layer by pyrolysis (3.1±0.3).

Besides the bare Pt, we also attempted to immobilize Pt clusters onto supporting materials as the control sample. The as-prepared Pt/cage sample was sonicated with graphite nanoplatelets and then pyrolyzed in air (with a low heating rate to 200°C) to remove the cage layer. However, the Pt clusters agglomerated upon removal of the cage during the pyrolysis process. Consequently, we chose commercial Pt/C as the control sample.

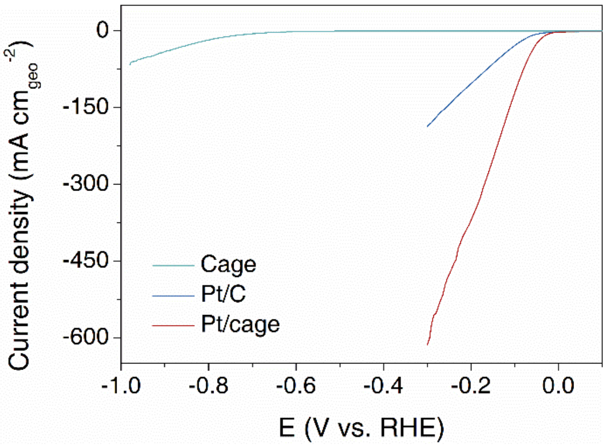

Supplementary Figure 7. Polarization curves comparison of cage, Pt/cage and Pt/C in 0.1 M KOH (pH = 13.00)

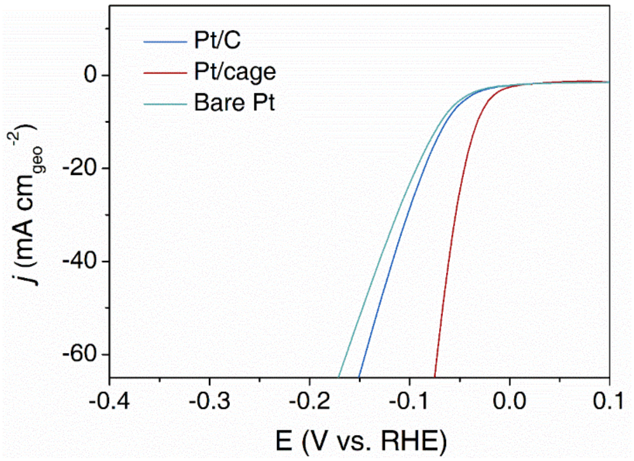

Supplementary Figure 8. Electrochemical HER performance of Pt without adding cage

The synthesized Pt without the addition of cage (referred to as 'bare Pt') formed Pt nanoparticles with an average size of 2.5 ± 0.3 nm and exhibited obvious agglomeration due to the lack of confinement provided by the cage (Supplementary Figure 3). The electrochemical hydrogen evolution performance of bare Pt was evaluated with the same Pt loading as Pt/C. As shown in **Figure S7**, the bare Pt demonstrated lower HER activity, with an overpotential of 69 mV at 10 mA cm^{-2}, compare to the commercial Pt/C, which had an overpotential of 64 mV at 10 mA cm^{-2}. This reduced activity is probably due to the agglomeration of Pt nanoparticles, which limits mass transport and reduces the availability of exposed Pt surface sites

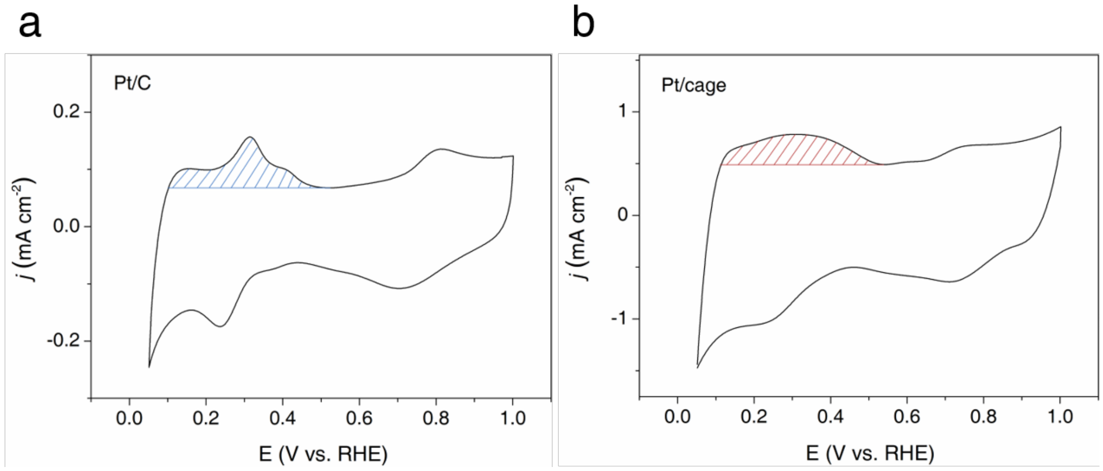

Supplementary Figure 9. Cyclic voltammetry (CV) of (a) Pt/C and (b) Pt/cage in Ar-saturated 0.1 M KOH.

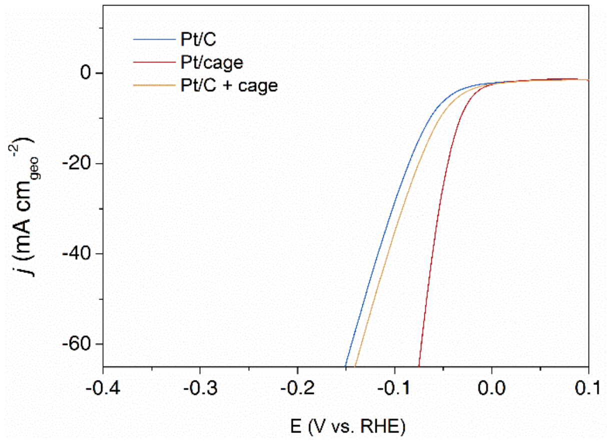

Supplementary Figure 10. Polarization curves comparison of Pt/C, Pt/cage, and Pt/C + cage in 0.1 M KOH (pH = 13.00)

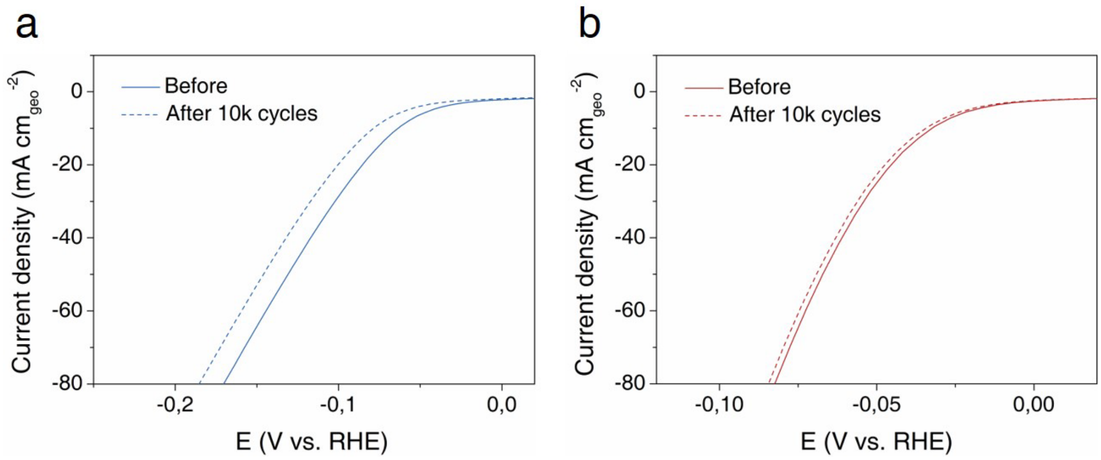

Supplementary Figure 11. Polarization curves comparison before and after 10k cycles on Pt/C and Pt/cage in 0.1 M KOH, respectively.

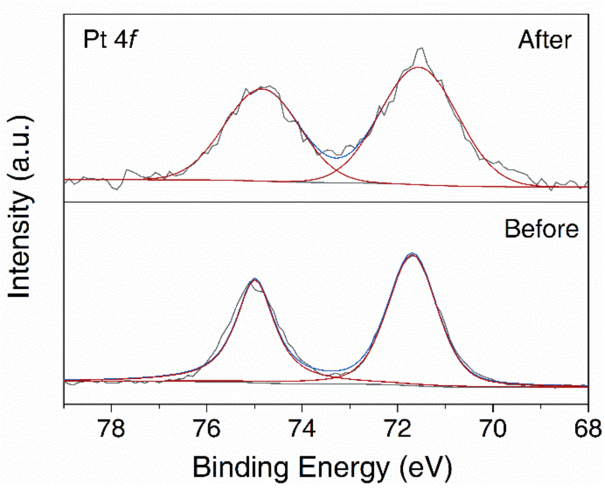

Supplementary Figure 12. X-ray photoelectron spectra of Pt/cage before and after 10k cycles.

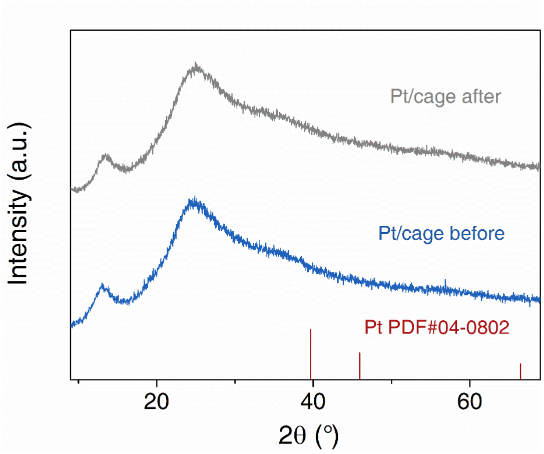

Supplementary Figure 13. X-ray diffraction pattern of Pt/cage before and after stability test.

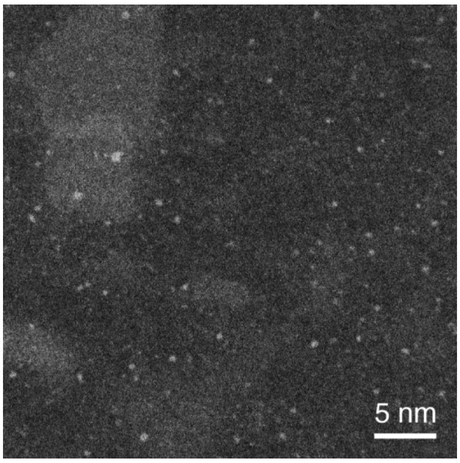

Supplementary Figure 14. HAADF-STEM image of Pt/cage after stability test.

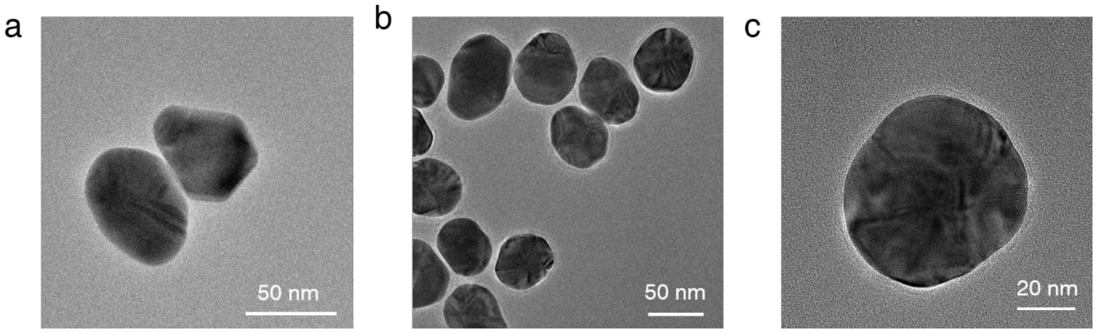

Supplementary Figure 15. (a) TEM image of the as-synthesized Au nanoparticles. (b) and (c) TEM image of the as-synthesized Au@SiO_2 SHINs (the average size is 55.4 ± 2.8 nm, and the average thickness of SiO_2 shell is ~2.0 nm).

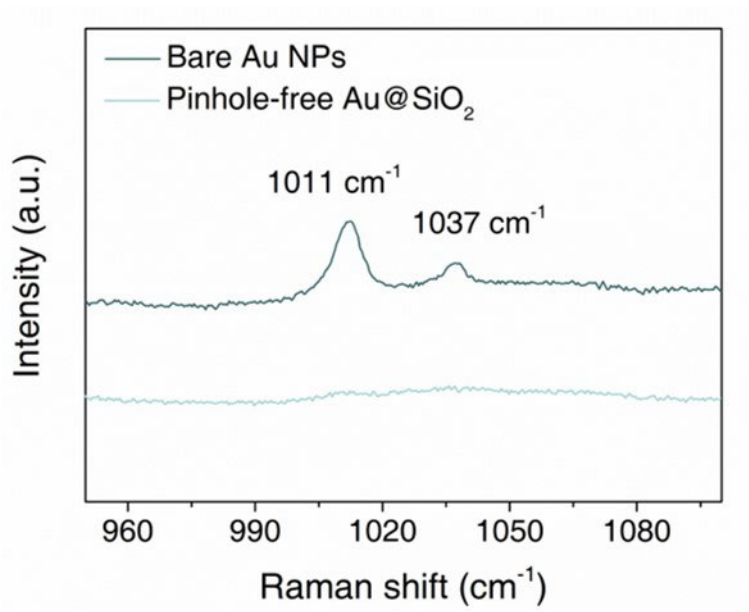

Supplementary Figure 16. Raman spectra of pyridine adsorbed on bare Au nanoparticles and pinhole-free Au@SiO_2 SHINs.

Pinhole test was conducted according to Li *et al*[8]. Briefly, 2μL of Au@SiO_2 shell-isolated nanoparticles (SHINs) solution on a clean Si wafer and dry it under Ar flow. 10 μL, 0.01mol L^{-1} of pyridine solution was dropped onto the SHINs-covered Si wafer, and let it absorbing for 2 min to ensure the pyridine molecules fully contact with the SHINs. SHINs with pinholes on the silica shell will allow the adsorption of pyridine molecules on Au core and results a strong Raman signal at the Raman shifts of 1011 cm^{-1} and 1037 cm^{-1}, corresponding to the ring bending vibrational modes of pyridine, while pinhole-free SHINs show no Raman signal for pyridine molecules.

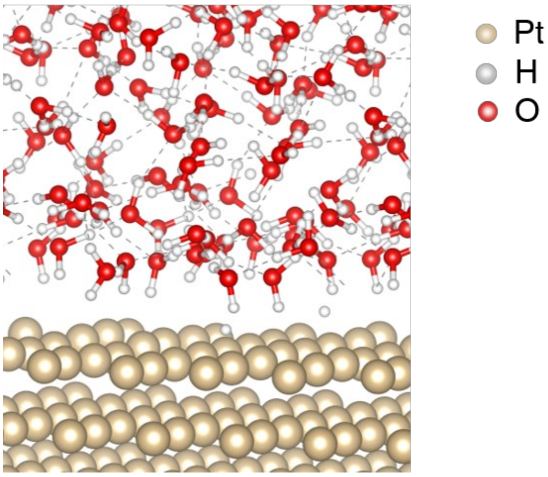

Supplementary Figure 17. Representative snapshot of the atomic configuration at Pt(100)-water interface at 300 K under alkaline condition, respectively. The corresponding colors for platinum atoms, hydrogen atoms, and oxygen atoms are yellow, white, and red, respectively.

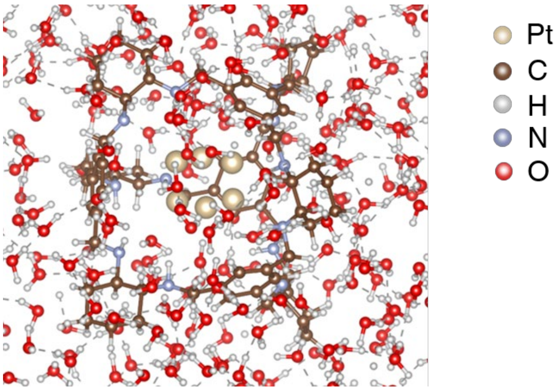

Supplementary Figure 18. Representative snapshot of the atomic configuration at Pt/cage-water interface at 300 K under alkaline condition, respectively. The corresponding colors for platinum atoms, carbon atoms, hydrogen atoms, nitrogen atoms, and oxygen atoms are yellow, brown, white, blue, and red, respectively.

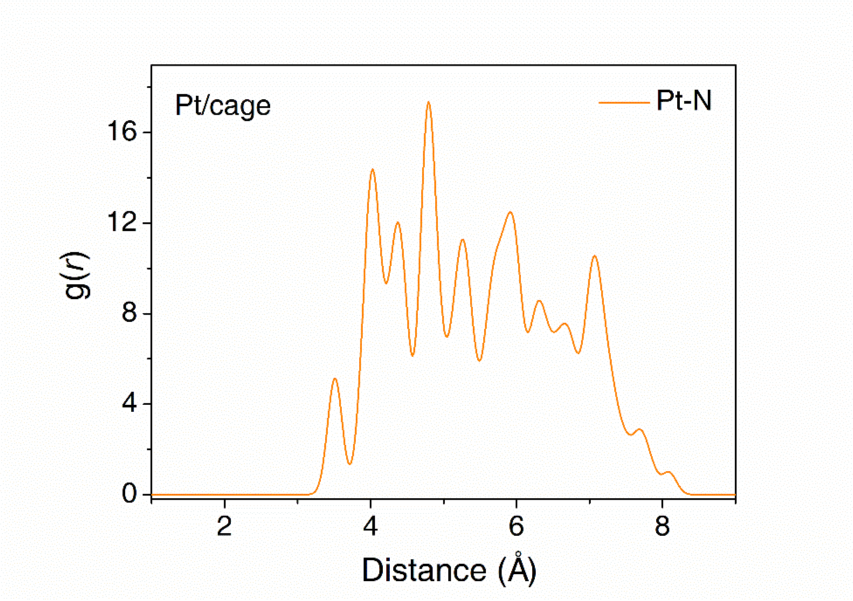

Supplementary Figure 19. Radial distribution function of N atom on Pt surface at the interface of Pt/cage-water.

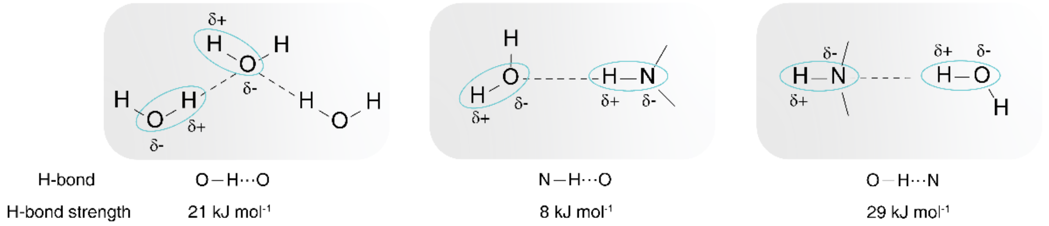

Supplementary Figure 20. Comparison of theoretical H-bond strength compare with the interfacial water net of Pt(100)-water and Pt/cage-water.

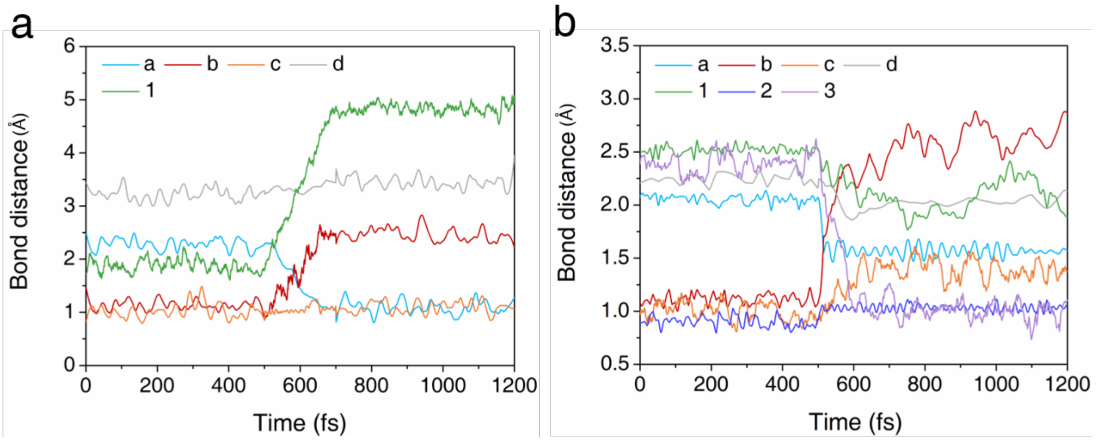

Supplementary Figure 21. (a) Bond distance evolution during the alkaline Volmer step at Pt(100)-water interface. (b) Bond distance evolution during the alkaline Volmer step at Pt/cage-water interface. The bonds correspond to the same bonds labeled in the schematic illustration in **Figures 4d and f**.

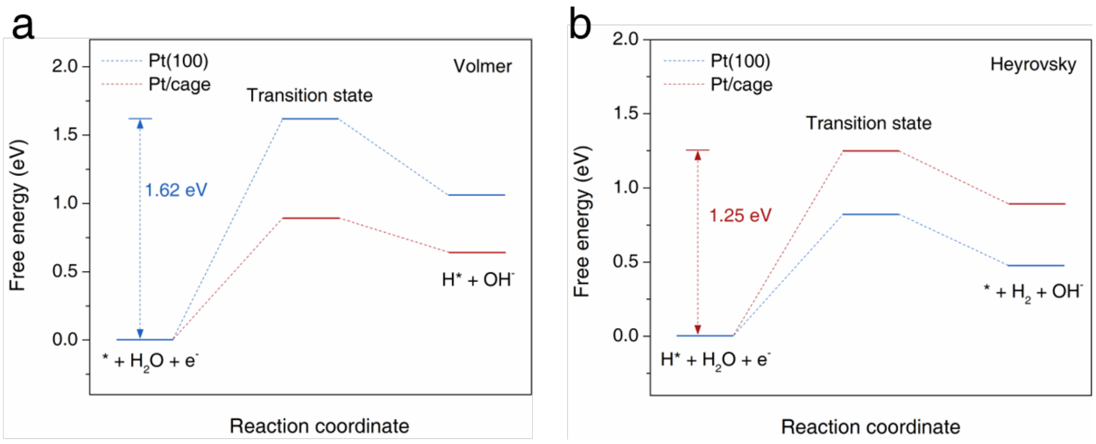

Supplementary Figure 22. Free energy diagrams of complete hydrogen evolution reaction on Pt(100) and Pt/cage. (a) Volmer step. (b) Heyrovsky step.

Following the Volmer step, Heyrovsky step was chosen as the second elementary step according the Tafel slope values in our experimental results which revealed that the hydrogen evolution reaction becomes Heyrovsky-limited on Pt/cage under alkaline condition. As it shown in **Supplementary Figure 22**, an energy barrier of 1.62 eV to overcome for the Volmer step on Pt(100), suggesting that the Volmer step is the rate-determining step of the whole hydrogen evolution reaction on Pt(100). However, this barrier is largely decreased and lower than that of Heyrovsky step (1.25 eV) on Pt/cage model, implying the easier Volmer step followed by the rate-determining Heyrovsky step on Pt/cage. The results were in good consistence with our experimental results that indicates an acid-like RDS and largely promoted hydrogen adsorption reaction with the manipulation by cage structure.

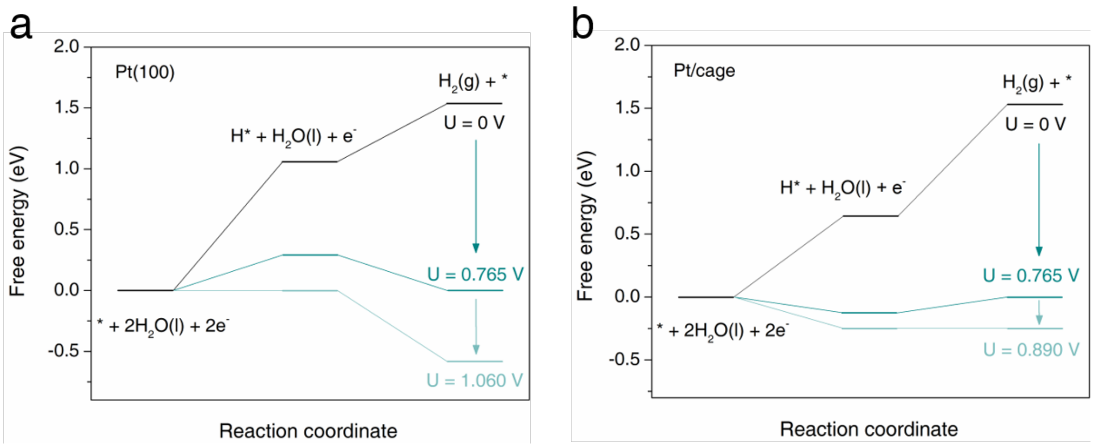

Supplementary Figure 23. Standard free energy diagram for HER on (a) Pt(100) and (b) Pt/cage at different electrode potential U.

As the **Supplementary Figure 23 a** shows, for hydrogen evolution reaction on Pt(100), the HER sub steps are all uphill when the electrode potential U is 0 V, which corresponds to a short-circuit state. At U = 0.765 V, the Volmer step remains uphill while the other elementary step becoming downhill. Only when the electrode potential increases to 1.06 V can all the elementary steps become downhill. Therefore, the information is obtained that the Volmer step is the rate-determining step and the theoretical overpotential is 1.060 V - 0.765 V = 0.295 V.

On Pt/cage (**Supplementary Figure 23 b**), at applied electrode potential U = 0.765 V, the Volmer step becomes downhill while Heyrovsky step remains uphill. All the elementary steps become downhill until the electrode potential increases to 0.890 V. This suggests that the first electron-transfer Volmer step is facilitated and Heyrovsky step becomes the rate-determining step. The overpotential is lowered to 0.890 V – 0.765 V = 0.125 V on Pt/cage, which is much lower than that on Pt(100) and consistent with our experimental results.

Supplementary tables

Supplementary Table 1. Results of the k^3-weighted Fourier transforms of Pt L_3-edge EXAFS spectra for Pt/cage, Pt foil, and PtO_2.

Sample	Peak in R space	Coordination confirmed by R space
Pt/cage	2.8 Å	Pt-Pt
Pt foil	2.8 Å	Pt-Pt
PtO_2	1.6 Å	Pt-O

Supplementary Table 2. Results of wavelet transformed Pt L_3-edge EXAFS spectra for Pt/cage, Pt foil, and PtO_2.

Sample	Contour intensity maximal in Wavelet transform	The scattering path confirmed by wavelet transform
Pt/cage	11.0 Å	Pt-Pt
Pt foil	11.0 Å	Pt-Pt
PtO_2	7.5 Å	Pt-O-Pt

Supplementary Table 3. Weight contents of Pt in as-prepared Pt/cage and bare Pt measured by ICP-OES.

Sample	Weight content
Pt/cage	5.3 wt.%
Pt/C	20.0 wt.%
Bare Pt	98.2 wt.%

Supplementary Table 4. The electrode geometrical area, mass loading, and ECSA of Pt/cage and Pt/C.

Sample	Geometrical area (cm^2)	Mass loading ($\mu g_{Pt}/cm_{geo}^{-2}$)	ECSA ($m^2\ g_{Pt}^{-1}$)
Pt/cage	0.1963	5.4	112.60
Pt/C	0.1963	5.1	71.33

Supplementary Table 5. Comparison of the Tafel slopes and overpotentials at 10 mA cm^{-2} with the state-of-art precious electrocatalysts.

Sample	Electrode	Temperature /°C	Electrolyte	η @10 mA cm_{geo}^{-2} (mV)	Tafel slope (mV dec^{-1})	Ref.
Pt/cage	Glassy carbon	25°C	0.1M KOH	32	37	This work
Pt/C (20 wt.%)	Glassy carbon	25°C	0.1M KOH	64	66	This work
$Pt_{3.21}Ni$ @Ti_3C_2	Glassy carbon	NA	0.1M KOH	55.6	39.5	9
1.9 nm Pt islands	Glassy carbon	NA	0.1M KOH	59	69	10
PtNi-O	Glassy carbon	20°C	1M KOH	39.8	78.8	11
Pt-Ni/C	Glassy carbon	NA	1M KOH	60	59	12
Pt_2Ni_3-P	Glassy carbon	NA	1M KOH	44	66	13
Ni_5P_4-Ru	Glassy carbon	NA	1M KOH	54	52	14
Pt/MgO	Glassy carbon	NA	1M KOH	39	39	15

Supplementary References

1 Henkelman, G. & Jónsson, H. Improved tangent estimate in the nudged elastic band method for finding minimum energy paths and saddle points. *J. Chem. Phys.* **113**, 9978-9985 (2000).

2 Henkelman, G., Uberuaga, B. P. & Jónsson, H. A climbing image nudged elastic band method for finding saddle points and minimum energy paths. *J. Chem. Phys.* **113**, 9901-9904 (2000).

3 Islam, S. M. R., Khezeli, F., Ringe, S. & Plaisance, C. An implicit electrolyte model for plane wave density functional theory exhibiting nonlinear response and a nonlocal cavity definition. *J. Chem. Phys.* **159**, 234117 (2023).

4 Skúlason, E. *et al.* Modeling the Electrochemical Hydrogen Oxidation and Evolution Reactions on the Basis of Density Functional Theory Calculations. *J. Phys. Chem. C* **114**, 18182-18197 (2010).

5 Man, I. C. *et al.* Universality in Oxygen Evolution Electrocatalysis on Oxide Surfaces. *ChemCatChem* **3**, 1159-1165 (2011).

6 Yang, F., Zhang, Q., Liu, Y. & Chen, S. A Theoretical Consideration on the Surface Structure and Nanoparticle Size Effects of Pt in Hydrogen Electrocatalysis. *J. Phys. Chem. C* **115**, 19311-19319 (2011).

7 Sun, F., Tang, Q. & Jiang, D.-e. Theoretical Advances in Understanding and Designing the Active Sites for Hydrogen Evolution Reaction. *ACS Catal.* **12**, 8404-8433 (2022).

8 Wang, Y.-H. *et al.* In situ electrochemical Raman spectroscopy and ab initio molecular dynamics study of interfacial water on a single-crystal surface. *Nat. Protoc.* **18**, 883-901 (2023).

9 Jiang, Y. *et al.* Coupling PtNi ultrathin nanowires with MXenes for boosting electrocatalytic hydrogen evolution in both acidic and alkaline solutions. *Small* **15**, 1805474 (2019).

10 Alinezhad, A. *et al.* Direct growth of highly strained Pt islands on branched Ni nanoparticles for improved hydrogen evolution reaction activity. *J. Am. Chem. Soc.* **141**, 16202-16207 (2019).

11 Zhao, Z. *et al.* Surface-engineered PtNi-O nanostructure with record-high performance for electrocatalytic hydrogen evolution reaction. *J. Am. Chem. Soc.* **140**, 9046-9050 (2018).

12 Kavian, R. *et al.* Pt-Ni octahedral nanocrystals as a class of highly active electrocatalysts toward the hydrogen evolution reaction in an alkaline electrolyte. *J. Mater. Chem. A* **4**, 12392-12397 (2016).

13 Wang, P., Shao, Q., Guo, J., Bu, L. & Huang, X. Promoting alkaline hydrogen evolution catalysis on P-decorated, Ni-segregated Pt-Ni-P nanowires via a synergetic cascade route. *Chem. Mater.* **32**, 3144-3149 (2020).

14 He, Q. *et al.* Achieving efficient alkaline hydrogen evolution reaction over a Ni_5P_4 catalyst incorporating single-atomic Ru sites. *Adv. Mater.* **32**, 1906972 (2020).

15 Tan, H. *et al.* Engineering a local acid-like environment in alkaline medium for efficient hydrogen evolution reaction. *Nat. Commun.* **13**, 2024 (2022).
