# Supplementary material for: Facilitating alkaline hydrogen evolution kinetics via interfacial modulation of hydrogen-bond networks by porous amine cages
Source: Nat Commun. 2025 Feb 21;16:1849. doi: 10.1038/s41467-025-56962-z (PMC11845474; doi:10.1038/s41467-025-56962-z)
Supplement: Supplementary file 2 — Description of Additional Supplementary Files [file 41467_2025_56962_MOESM2_ESM.pdf]

Description of Supplementary Data files

Supplementary Data 1
Raw data of the Pt(100)-water interface configuration
Raw data of the Pt/cage-water interface configuration
